# The ribosome-associated quality control pathway supports survival in the absence of non-stop ribosome rescue factors

**DOI:** 10.1128/mbio.02322-24

**Published:** 2024-11-13

**Authors:** Katrina Callan, Cassidy R. Prince, Heather A. Feaga

**Affiliations:** 1 Department of Microbiology, Cornell University, Ithaca, New York, USA; University of Washington School of Medicine, Seattle, Washington, USA

**Keywords:** ribosomes, translation, *trans*-translation, bacteria, molecular genetics, RNA binding proteins

## Abstract

In bacteria, if a ribosome translates an mRNA lacking a stop codon it becomes stalled at the 3′ end of the message. These ribosomes must be rescued by *trans*-translation or the alternative rescue factors (ArfA or ArfB). However, mounting evidence suggests that the ribosome quality control (RQC) pathway may also rescue non-stop ribosomes. Here, we surveyed the conservation of ribosome rescue pathways in >15,000 bacterial genomes. We found that *trans*-translation is conserved in >97% of bacterial genomes, while the other rescue pathways are restricted to particular phyla. We did not detect the gene encoding RqcH, the major mediator of RQC, in Proteobacteria (Pseudomonadota). In all Proteobacteria investigated to date, *trans*-translation is essential in the absence of the Arf proteins. Therefore, we tested whether expression of RQC components from *Bacillus subtilis* could rescue viability in the absence of *trans*-translation and ArfA in *Escherichia coli*. We found that the RQC pathway indeed functions in *E. coli* and rescues the well-documented synthetic lethal phenotype of ∆*ssrA*∆*arfA*. Moreover, we show that the RQC pathway in *B. subtilis* is essential in the absence of *trans*-translation and ArfA, further supporting a role for the RQC pathway in the rescue of non-stop ribosomes. Finally, we report a strong co-occurrence between RqcH and the ribosome splitting factor MutS2, but present experimental evidence that there are likely additional ribosome splitting factors beyond MutS2 in *B. subtilis*. Altogether, our work supports a role for RQC in non-stop ribosome rescue and provides a broad survey of ribosome rescue pathways in diverse bacteria.

**IMPORTANCE:**

In bacteria, it is estimated that 2%–4% of all translation reactions terminate with the ribosome stalled on a damaged mRNA lacking a stop codon. Mechanisms that rescue these ribosomes are essential for viability. We determined the functional overlap between the ribosome quality control pathway and the classical non-stop rescue systems [alternative rescue factor (ArfA) and *trans*-translation] in a representative Firmicute and Proteobacterium, phyla that are evolutionarily distinct. Furthermore, we used a bioinformatics approach to examine the conservation and overlap of various ribosome rescue systems in >15,000 species throughout the bacterial domain. These results provide key insights into ribosome rescue in diverse phyla.

## INTRODUCTION

Truncated mRNAs that lack stop codons (non-stop mRNAs) are a major problem in bacteria (1, 2). The release factors (RF1 and RF2) require stop codon recognition to terminate translation (3, 4). Therefore, if an mRNA lacks a stop codon, the ribosome becomes stalled at the 3′ end of the message (5). Non-stop mRNAs arise from premature transcription termination, mRNA degradation by nucleases, and stop codon readthrough. Bacteria have evolved ways to rescue ribosomes from non-stop mRNAs. The primary rescue system, *trans*-translation, is mediated by transfer-messenger RNA (tmRNA) encoded by the *ssrA* gene and its partner protein, small protein B (SmpB) (5, 6). SmpB bound to tmRNA senses the empty mRNA channel of the non-stop ribosome, and translation then resumes on the mRNA-like domain of tmRNA (7, 8). A degradation tag, encoded by the short reading frame of tmRNA, is added to the nascent peptide that will target the truncated protein for proteolysis (5, 9, 10). A survey of 2,031 bacterial genomes did not identify any species that lacked *ssrA* and *smpB*, suggesting that *trans*-translation is completely conserved in all bacteria (11). Moreover, *trans*-translation is essential in many species, including *Mycobacterium tuberculosis*, *Shigella flexneri*, and *Neisseria gonorrhoeae* (12–14).

An alternative non-stop rescue factor, ArfA, was uncovered in *Escherichia coli* in a screen for genes that are essential for viability in cells lacking *ssrA* (15). ArfA recognizes the empty mRNA channel and recruits RF2 to terminate translation independent of a stop codon (16–18). Since either ArfA or *trans*-translation is necessary for survival, it is clear that *E. coli* needs at least one mechanism for rescuing non-stop ribosomes. A second alternative rescue factor, ArfB, is essential for viability in *Caulobacter crescentus* in the absence of *trans*-translation (19). *C. crescentus* does not encode ArfA. Although *E. coli* encodes ArfB, it is poorly expressed and is sufficient to rescue the synthetic lethality of ∆*ssrA*∆*arfA* only when expressed from an inducible promoter (20). The essentiality of *trans*-translation in many bacteria and the conditional essentiality of ArfA and ArfB in the absence of *trans*-translation indicate that rescuing ribosomes from truncated mRNAs is essential for cell survival (21).

*Bacillus subtilis* can survive without *trans*-translation and encodes an alternative rescue factor called *Bacillus* ribosome rescue factor A (BrfA) (22). *B. subtilis* 168 BrfA is homologous to *E. coli* MG1655 ArfA, sharing 21.54% protein sequence identity when aligned with Clustal Omega (23). Like ArfA, BrfA recognizes non-stop ribosomes and recruits RF2 to terminate translation (22). BrfA is also negatively regulated by *trans*-translation, since the *brfA* transcript contains an RNase III cleavage site upstream of the stop codon and is therefore translated from a non-stop mRNA (22). Depletion of SmpB from ∆*brfA B. subtilis* results in a severe synthetic growth defect, but is not lethal, suggesting that non-stop ribosomes can still be rescued in this strain (22).

*B. subtilis* also uses the ribosome quality control (RQC) pathway to rescue stalled ribosomes (24–27). In this pathway, ribosome stalling and collisions trigger the recruitment of a ribosome splitting factor to split the ribosome from the mRNA in an ATP-dependent manner (28, 29). The peptidyl-tRNA remains bound to the large ribosomal subunit. Next, RqcH and its cofactor (either RqcP or YlmH) recruit alanine-charged tRNAs to the large subunit and catalyze the addition of an alanine tag to the stalled peptide in a template-independent manner (24, 27). The alanine tail serves as a degron tag to target the stalled peptide for degradation (24, 26). Additionally, the alanine tail exposes the aminoacyl bond between the stalled peptide and the tRNA to the cytoplasm, where peptidyl-tRNA hydrolase (PTH) can hydrolyze this bond and thus, free the nascent chain from the tRNA (30). The large subunit is then available for a new round of translation.

Here, we show that components of the RQC pathway from *B. subtilis* are sufficient to rescue *E. coli* cells lacking both *trans*-translation and the alternative rescue factor ArfA. We further show that *trans*-translation becomes essential in *B. subtilis* in the absence of both the ArfA homolog BrfA and the RQC pathway. These data support a role for RQC in rescuing non-stop ribosomes and highlight the importance of this rescue for maintaining cell viability. We also report a bioinformatic analysis of >15,000 genomes across the bacterial domain to determine the prevalence and distribution of *trans*-translation, ArfA/BrfA, ArfB, and RqcH. We find that genes encoding *trans*-translation are present in >97% of bacterial genomes. We also report a strong co-occurrence between RqcH and the ATP-dependent ribosome splitting factor MutS2. However, experimentally our results suggest that there are additional ribosome splitting factors in *B. subtilis*. Our findings provide a comprehensive view of the conservation and functional interaction between non-stop ribosome rescue systems.

## RESULTS

### The distribution of ribosome rescue pathways in bacteria reveals varying strategies for non-stop ribosome rescue

**Fig 1:**
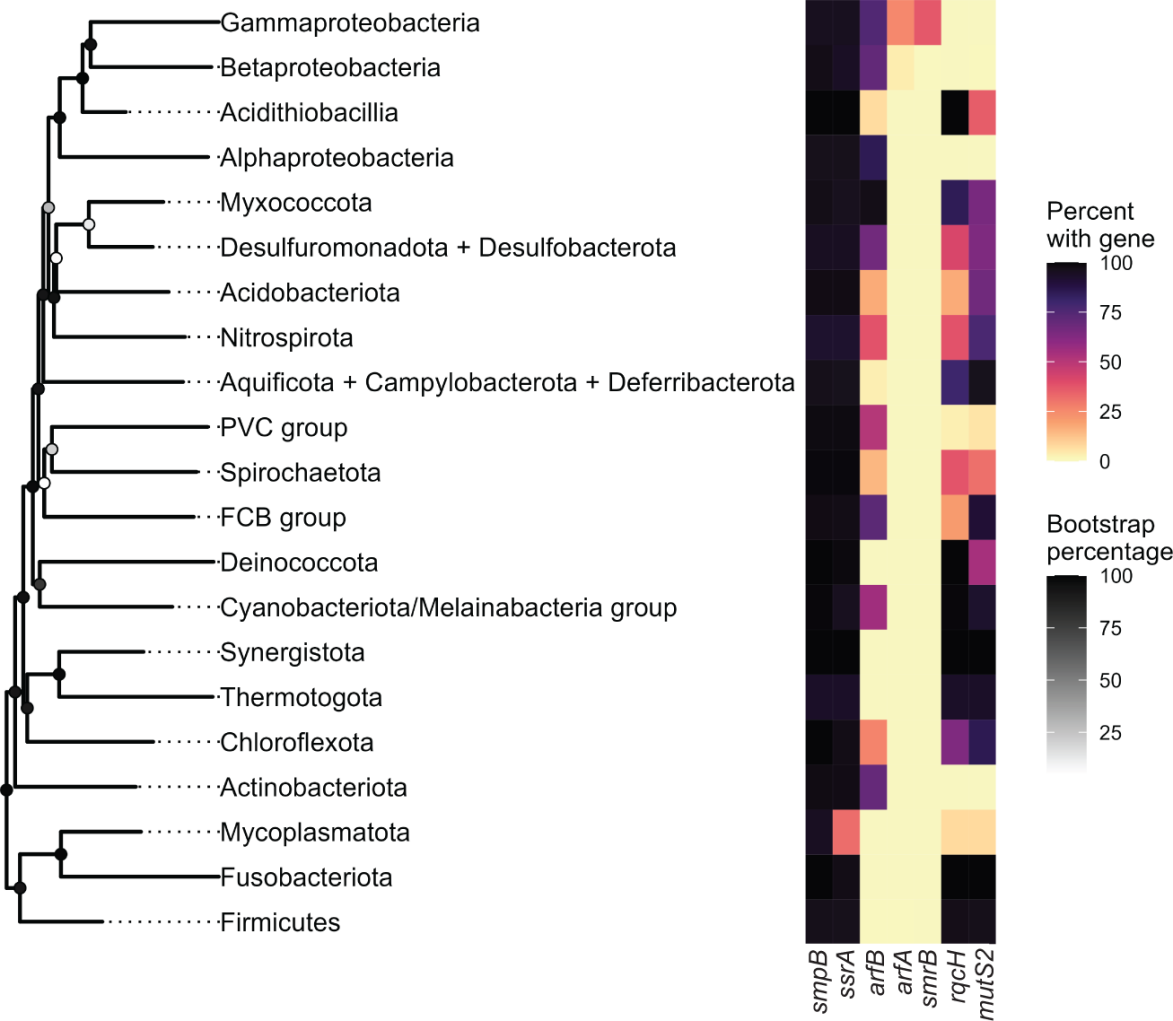
Conservation of ribosome rescue pathways across >15,000 bacterial genomes. Heatmap showing the percentage of genomes per phyla containing *smpB*, *ssrA*, *arfA*, *arfB*, *smrB*, *rqcH*, and *mutS2*. The tree was built using 16S rRNA genes from a single representative species for each phylum (Table 2). NCBI accession numbers of all surveyed genomes are given in Table S1, and the query sequences used to search for each gene are given in Table S2. Bootstrap percentages are shown as darkened circles at each node.

To investigate the conservation and distribution of ribosome rescue pathways in bacteria, we surveyed 15,259 representative reference genomes for the presence of genes encoding the canonical non-stop rescue factors: tmRNA, SmpB, ArfA, and ArfB. We detected the genes encoding *trans*-translation in nearly all bacterial genomes (Fig. 1; Table 1; Table S1). *smpB* was detected in 97% of the representative genomes surveyed, and *ssrA* was detected in 96%. Consistent with previous reports, genomes in which we did not detect *ssrA* were found mainly in Mycoplasmatota, suggesting a major loss event within that phylum (11). However, most Mycoplasmatota genomes still contain *smpB*, indicating that *ssrA* may be present but has not been detected. *ssrA* is more difficult to detect than *smpB* since some bacteria, such as *C. crescentus*, encode a circularly permutated tmRNA that requires RNase processing (31). It is possible that Mycoplasmatota species encode tmRNA with a permutation that has not yet been identified and characterized.

**TABLE 1:** Conservation of ribosome rescue pathways across 15,259 genomes

Phylum	Total genomes	*smpB*	*ssrA*	*arfB*	*arfA*	*smrB*	*rqcH*	*mutS2*
Acidithiobacillia	14	14	14	1	0	0	0	5
Acidobacteriota	46	45	45	8	0	0	8	31
Actinobacteriota	3,213	3,148	3,141	2,245	1	0	0	0
Alphaproteobacteria	2,376	2,296	2,293	2,022	0	0	0	0
Aquificota + Campylobacterota + Deferribacterota	191	185	184	6	0	0	153	184
Armatimonadota	3	3	3	1	0	0	2	3
Bdellovibrionota	6	6	6	5	0	0	5	5
Betaproteobacteria	1,000	975	943	709	37	2	0	4
Caldisericota/Cryosericota group	1	1	0	0	0	0	0	1
Chloroflexota	41	41	40	11	0	0	26	35
Coprothermobacterota	2	2	2	0	0	0	2	2
Cyanobacteriota/Melainabacteria group	162	161	155	90	0	0	161	150
Deinococcota	101	101	100	0	0	0	101	55
Deltaproteobacteria	6	6	6	4	0	0	6	6
Desulfuromonadota + Desulfobacterota	185	176	176	124	0	0	77	118
Dictyoglomota	2	2	2	0	0	0	2	2
Fibrobacterota, Chlorobiota, and Bacteroidota (FCB group)	1,956	1,913	1,910	1,426	0	0	404	1,781
Firmicutes	2,633	2,551	2,541	5	5	0	2,557	2,550
Fusobacteriota	42	42	41	0	0	0	42	42
Gammaproteobacteria	2,704	2,588	2,588	2,027	699	1,004	0	1
Mycoplasmatota	119	113	38	0	0	0	9	9
Myxococcota	72	70	69	70	0	0	61	47
Nitrospinae/Tectomicrobia group	2	2	2	0	0	0	0	2
Nitrospirota	13	12	12	5	0	0	5	10
Planctomycetota, Verrucomicrobiota, and Chlamydiota (PVC group)	211	208	208	107	0	0	7	12
Spirochaetota	108	107	107	16	0	0	15	15
Synergistota	15	15	15	0	0	0	15	15
Thermotogota	31	29	29	0	0	0	29	29
Zetaproteobacteria	4	4	4	4	0	0	0	0
Total	15,259	14,816	14,674	8,886	742	1,006	3,727	5,133

**TABLE 2:** Strains, plasmids, primers, and representative genomes for 16S tree

Strain, plasmid, primer, or genome	Description, sequence, or accession no.	Source
Strains		
HAF1	168 *trpC2 B. subtilis* wild type	(32)
KC118	168 *trpC2* ∆*rqcH*::kan	This study
KC145	168 *trpC2 ∆smpB*	This study
KC153	168 *trpC2 ∆smpB∆rqcH*::kan	This study
KC158	168 *trpC2* ∆*brfA*	
KC167	168 *trpC2 ∆brfA∆rqcH*::kan	This study
HRH774	168 *trpC2 lacA*::P_xyl_-*dCas9*-mls	(33)
KC357	168 *trpC2 lacA*::P_xyl_-*dCas9*-mls *amyE*::P_veg_-sgRNA*^smpB^*	This study
KC379	168 *trpC2 ∆brfA∆mutS2::*kan *lacA*::P_xyl_-*dCas9*-mls *amyE*::P_veg_-sgRNA*^smpB^*	This study
KC381	168 *trpC2 ∆rqcH:*:kan *lacA*::P_xyl_-*dCas9*-mls *amyE*::P_veg_-sgRNA*^smpB^*	This study
KC383	168 *trpC2 ∆brfA∆rqcH::*kan *lacA*::P_xyl_-*dCas9*-mls *amyE*::P_veg_-sgRNA*^smpB^*	This study
KC385	168 *trpC2 ∆brfA lacA*::P_xyl_-*dCas9*-mls *amyE*::P_veg_-sgRNA*^smpB^*	This study
KC462	168 *trpC2 ∆mutS2:*:kan *lacA*::P_xyl_-*dCas9*-mls *amyE*::P_veg_-sgRNA*^smpB^*	This study
KCK19	MG1655 WT	(34)
KCK18	MG1655∆*ssrA*::cat	(34)
KCK 345	BW25113 ∆*arfA*::kan (Keio collection)	(35)
KC444	MG1655∆*ssrA*::cat∆*arfA*::kan pBAD322-RqcH-RqcP	This study
Plasmids		
pDR244	Cre-lox plasmid	(36)
pJMP1	pJMP1 *lacA*::P_xyl_-*dCas9*	(37)
pKC349	pJMP2 *amyE*::P_veg_-sgRNA*^smpB^*	(22)
pOPO646	pBAD322-arapBAD	DQ119282.1
pKC353	pBAD322-*rqcH*	This study
pKC426	pBAD322-*rqcH*-*rqcP*	This study
Primers		
sp60	5′-GCTCGTGTTGTACAATAAATGTAGGAATCCTTAAGGTTTACGGTTTTAGAGCTAGAAATAGCAAGTTAAAATAAGGC-3′	(22)
HRH175	5′-ACATTTATTGTACAACACGAGCC-3′	(33)
HAF1	5′-CTACCGCTTTGACGAACAGCATTTCG-3′	This study
HAF2	5′-GATATGCCTGAAACAGCTCATTGCAG-3′	This study
brfAchkF	5′-GCACGGAGATTAATCATGATATGC-3’	This study
brfAchkR	5′-CTGGCTGATGGTCATACTGTTGAC-3′	This study
KC31	5′-TCGTTTGGTCCATGCTTGTC-3′	This study
KC32	5′-GAAAGAGCTTGCTGAAAACGTGG-3′	This study
KC53	5′-TGGGCTAGCAGGAGGAATTCATTGCATATGTCGTTTGATGGCATGTTTACATAC-3′	This study
KC54	5′-GGATCCCCGGGTACCATGGTCAGCTTTTTTTGAGCTTGATGACAGTATCAGC-3′	This study
MG1655_ssrA_F	5′-CTGGTCATGGCGCTCATAAATCTGGTATAC-3′	This study
MG1655_ssrA_R	5′-TCGGATGACTCTGGTAATCACCGATGGAG-3′	This study
MG1655_arfA_F	5′-GTTGTTGATTTTTTGCACTGGCAGG-3′	This study
MG1655_arfA_R	5′-ATTCGTGATTTGCTGAAAGAGCAGAATAAC-3′	This study
KC61	5′-TCGCAACTCTCTACTGTTTCTCCATACCCG-3′	This study
KC62	5′-GAAAATCTTCTCTCATCCGCCAAAACAGCC-3′	This study
Genomes		
Acidithiobacillia	GCF_003721225.1	
Acidobacteriota	GCF_004123295.1	
Actinobacteriota	GCF_012911925.1	
Alphaproteobacteria	GCF_001633145.1	
Aquificota + Campylobacterota + Deferribacterota	GCF_000421105.1	
Betaproteobacteria	GCF_007830455.1	
Chloroflexota	GCF_000025005.1	
Cyanobacteriota/ Melainabacteria group	GCF_000317105.1	
Deinococcota	GCF_003336745.1	
Desulfuromonadota + Desulfobacterota	GCF_014773365.1	
FCB group	GCF_002894645.1	
Firmicutes	GCF_000763575.1	
Fusobacteriota	GCF_900167275.1	
Gammaproteobacteria	GCF_003569005.1	
Mycoplasmatota	GCF_016865445.1	
Myxococcota	GCF_012933655.1	
Nitrospirota	GCF_900169565.1	
PVC group	GCF_016595505.1	
Spirochaetota	GCF_000332515.2	
Synergistota	GCF_000526375.1	
Thermotogota	GCF_003990895.1	

Of the alternative rescue factors, *arfB* is widely distributed across the bacterial domain and is conserved in 58% of our surveyed genomes. The ArfB homolog ICT1 rescues non-stop mitochondrial ribosomes, suggesting it was present in the mitochondrial progenitor (38–40). Most Gram-positive phyla, including Firmicutes, lack *arfB*. The alternative rescue factor ArfA was the least conserved rescue mechanism and is restricted specifically to Gammaproteobacteria, some Betaproteobacteria, and 10 species in the *Bacillus subtilis* group.

Next, we surveyed the distribution and conservation of *rqcH* to determine the prevalence of the RQC pathway. We detected *rqcH* in 24% of genomes distributed across almost every phylum in the bacterial domain, consistent with the idea that it was present in the last universal common ancestor (29) (Table S1). We did not detect *rqcH* in any Proteobacteria. All Proteobacteria studied to date require either *trans*-translation or one of the alternative non-stop ribosome rescue factors for viability (13, 15, 19). Therefore, deletion of the genes encoding *trans*-translation and the Arf proteins may be synthetically lethal in Proteobacteria because these organisms lack the RQC pathway.

### *B. subtilis* RQC machinery functions in *E. coli* and restores viability in the absence of *trans*-translation and ArfA

**Fig 2:**
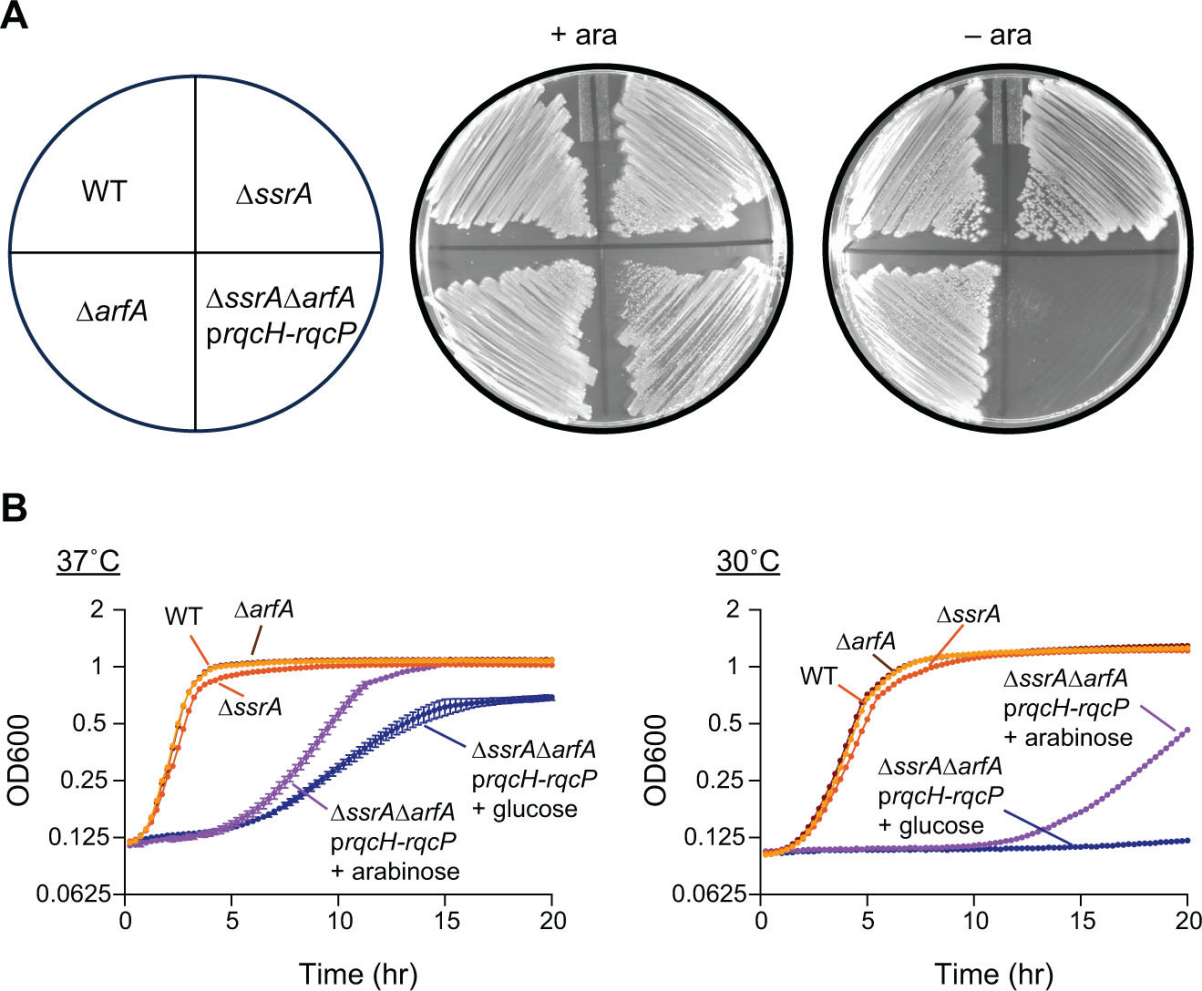
*E. coli* can survive the synthetic lethality of ∆*ssrA::cat^R^*∆*arfA::kan^R^* when provided with *B. subtilis rqcH* and *rqcP*. (**A**) Strains depicted in the schematic were grown on plates with or without 1% arabinose to induce expression of RqcH/RqcP. (**B**) Growth curves in lysogeny broth (LB) of *E. coli* ∆*arfA::kan^R^*, ∆*ssrA::cat^R^*, and ∆*ssrA::cat^R^*∆*arfA::kan^R^* strain harboring the plasmid expressing RqcH and RqcP. Error bars represent the standard deviation of three independent growth curves performed in parallel from individual colonies.

We hypothesized that the RQC pathway could functionally substitute for non-stop ribosome rescue pathways if expressed in Proteobacteria and that the synthetic lethal phenotype of ∆*ssrA*∆*arfA* could be rescued by this pathway. To test this, we constructed a plasmid encoding *rqcH* and its partner protein *rqcP* under the control of an arabinose inducible promoter (p*rqcH*-r*qcP*). We transformed this plasmid into *E. coli* MG1655∆*ssrA::cat^R^* and then transduced the *∆arfA::kan^R^* deletion into this strain. Colonies were recovered on plates containing kanamycin and 1% arabinose. The presence of the ∆*ssrA::cat^R^*∆*arfA::kan^R^* double deletion was confirmed by PCR. These cells did not grow on plates in the absence of arabinose, indicating that expression of RqcH and RqcP is required for survival in this condition (Fig. 2A). As expected, when ∆*arfA::kan^R^* was transduced into the ∆*ssrA:cat^R^* strain, no colonies were recovered. In liquid culture, ∆*ssrA::cat^R^*∆*arfA::kan^R^* + p*rqcH*-*rqcP* grew better in the presence of arabinose (Fig. 2B), although growth was not recovered to wild-type levels. These results demonstrate that RqcH and RqcP are sufficient to support *E. coli* survival when *trans-*translation and ArfA are absent and support a role for the RQC pathway in non-stop ribosome rescue.

### *trans*-Translation is required for cell growth in the absence of *brfA* and the RQC pathway in *B. subtilis*

**Fig 3:**
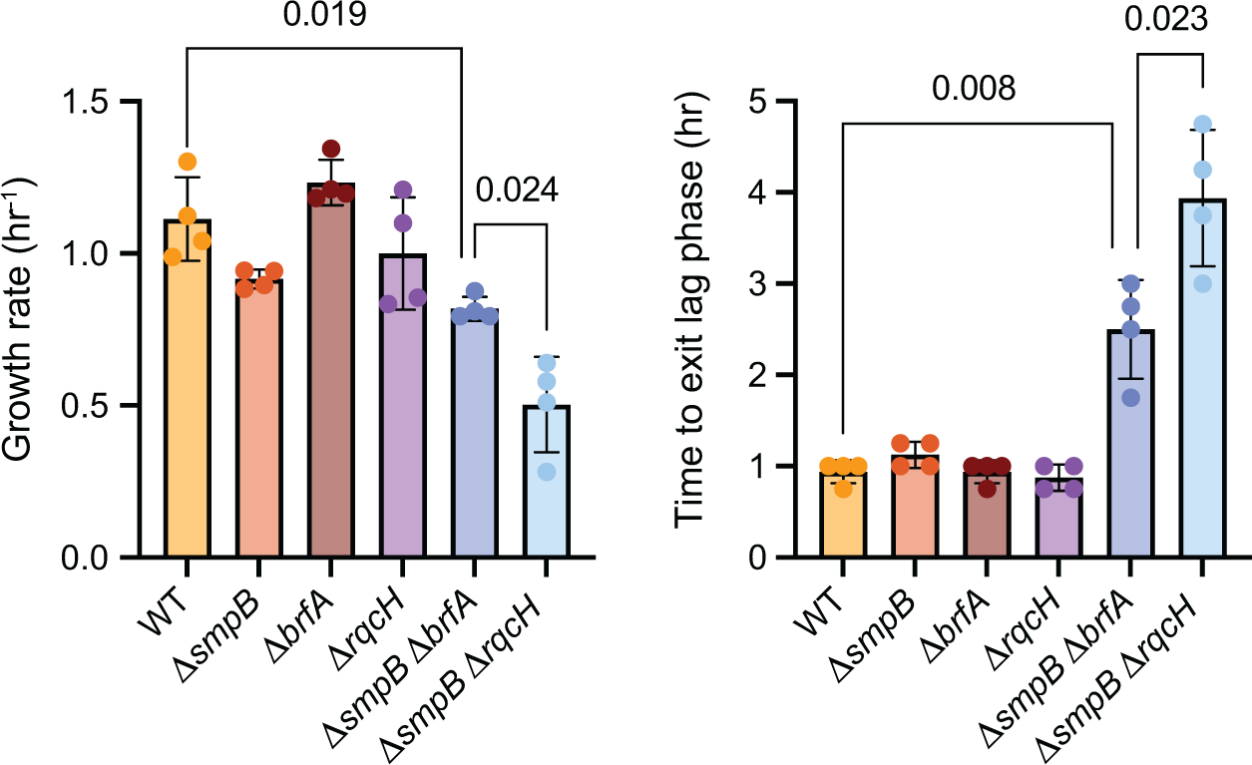
*Bacillus subtilis* ∆*smpB*∆*rqcH* and ∆*smpB*∆*brfA* cells exhibit defects in maximum growth rate and delayed entry into the exponential phase. Cells were grown under aerobic conditions in LB at 37°C. (Left) Maximum growth rate. (Right) Time to exit lag phase (measured as time at which optical density exceeds 0.125 when starting from 0.05 OD diluted from overnight culture). Error bars represent the standard deviation of four independent replicates performed on different days. *P* values represent the results of an unpaired *t*-test with Welch’s correction.

*B. subtilis* lacking *trans*-translation exhibits a severe fitness defect when either *brfA* or *rqcH* is deleted (22, 28). We further characterized the growth rate and time to exit lag phase in ∆*smpB*∆*brfA* and ∆*smpB*∆*rqcH* in *B. subtilis* (Fig. 3). When comparing ∆*smpB*∆*rqcH* to ∆*smpB*∆*brfA*, the ∆*smpB*∆*rqcH* strain exhibited a significantly slower maximal growth (*P* = 0.024) and significantly longer lag phase (*P* = 0.023). These data suggest that when *B. subtilis* lacks *trans*-translation, the RQC pathway greatly contributes to cellular fitness, even more so than BrfA under the conditions we examined.

**Fig 4:**
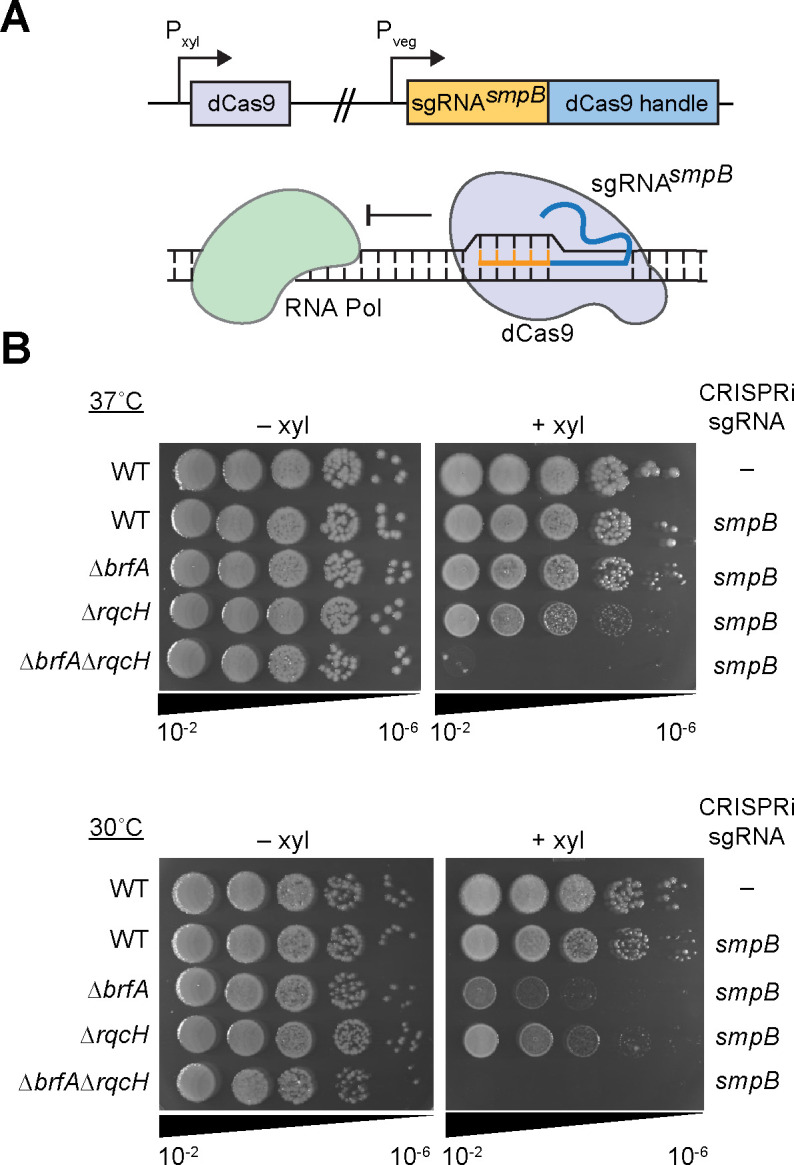
Impact of SmpB depletion from cells lacking *brfA* and *rqcH* in *B. subtilis*. (**A**) Schematic showing depletion of *smpB* using CRISPRi. Guide RNA targeting *smpB* (sgRNA*^smpB^*) under the vegetative promoter in *B. subtilis* is co-expressed with deactivated Cas9 (*dCas9*) under the control of a xylose inducible promoter resulting in transcriptional repression. (**B**) Strains harboring sgRNA*^smpB^* and *dCas9* were serially diluted and spot plated on LB agar with or without 1% xylose at 37°C and 30°C. Images are representative of three independent experiments.

To our knowledge, the impact of losing all three rescue systems in *B. subtilis* has not been reported. Therefore, we used CRISPR interference (CRISPRi) (37, 41) to deplete SmpB from cells lacking both *rqcH* and *brfA*. We expressed nuclease deficient Cas9 (dCas9) under the control of a xylose-inducible promoter while constitutively expressing a guide RNA targeting *smpB* (sgRNA*^smpB^*) (Fig. 4A). When *smpB* expression was blocked in the ∆*rqcH∆brfA* strain, colonies failed to form on plates at 37°C (Fig. 4B). These results indicate that *trans*-translation becomes essential in the absence of BrfA and RqcH, further supporting a role for the RQC pathway in rescuing non-stop ribosomes.

Interestingly, we observe that SmpB depletion from ∆*brfA* cells has a greater impact on fitness than depletion from ∆*rqcH* cells, suggesting that the RQC pathway may not function as well for rescue at the lower temperature of 30˚C (Fig. 4). These results are in agreement with the RqcH-RqcP complementation in *E. coli*, where expression of RqcH-RqcP did not rescue ∆*ssrA*∆*arfA* cells as well at 30°C as at 37°C (Fig. 2B).

### RqcH ribosome rescue activity is decreased, but not abolished, in the absence of MutS2

**Fig 5:**
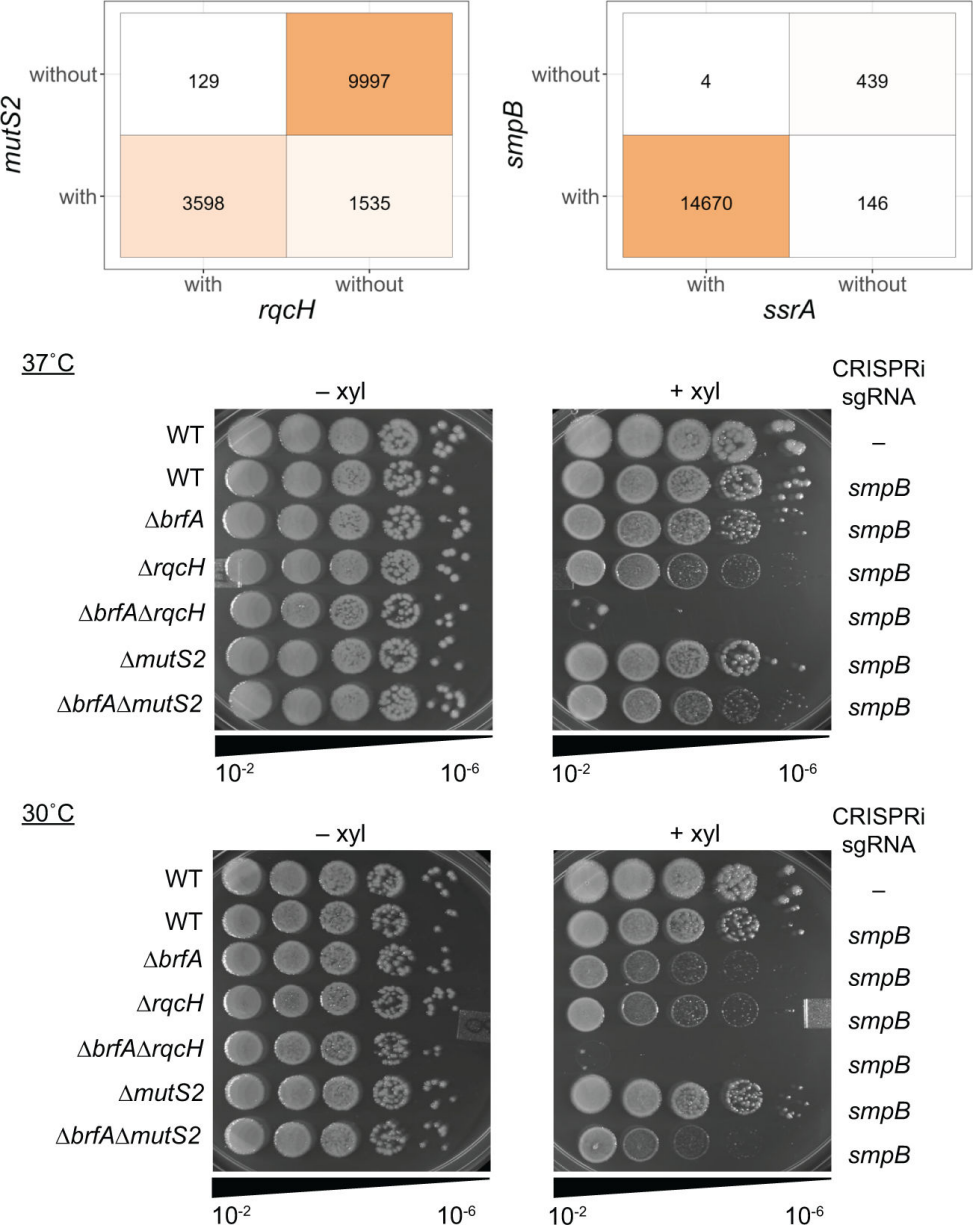
The RQC pathway can still function in the absence of MutS2. (Top) Co-occurrence matrices of *mutS2* vs *rqcH* and *smpB* vs *ssrA*. The values represent the number of genomes with or without these genes. (Bottom) Strains harboring sgRNA*^smpB^* and *dCas9* were serially diluted and spot plated on LB agar with or without 1% xylose at 37°C and 30°C. Image shows representative from triplicate experiments.

RqcH cannot act directly on non-stop ribosomes but requires ribosomal subunits to be separated from the mRNA before it can perform its tagging function. MutS2 is a recently discovered ribosome splitter in *B. subtilis* (28, 29). Genes that function in the same pathway exhibit a high degree of co-occurrence. For example, *ssrA* and *smpB* exhibit near complete co-occurrence (Fig. 5). Therefore, we investigated how frequently MutS2 and RqcH co-occur in bacterial genomes. Of the genomes that encode RqcH, 97% also encode MutS2 (Fig. 5). Of the genomes that have lack RqcH, 87% have also lack MutS2. Consistent with previous reports, these data indicate that there is a high degree of co-occurrence between RqcH and MutS2 and strongly suggest that they function in the same pathway (28).

If MutS2 is the only ribosome splitter capable of generating substrates for RqcH, then *mutS2* deletion phenotypes should be similar in severity to *rqcH* deletion phenotypes. To test this, we compared the impact of SmpB depletion from ∆*brfA*∆*rqcH* cells to SmpB depletion from ∆*brfA*∆*mutS2* cells. In the ∆*brfA* background, when SmpB is depleted, cells are totally reliant on RqcH. In three independent experiments, SmpB depletion from ∆*brfA*∆*mutS2* exhibited a more severe fitness defect than SmpB depletion from the ∆*brfA* single deletion, indicating that MutS2 is a major splitting factor acting upstream of RqcH (Fig. 5). However, this fitness defect was not as severe as SmpB depletion from ∆*brfA*∆*rqcH*. These data indicate that RqcH retains some function in the absence of MutS2 and that there may be additional factors capable of splitting ribosomes from non-stop messages prior to rescue by RqcH.

## DISCUSSION

**Fig 6:**
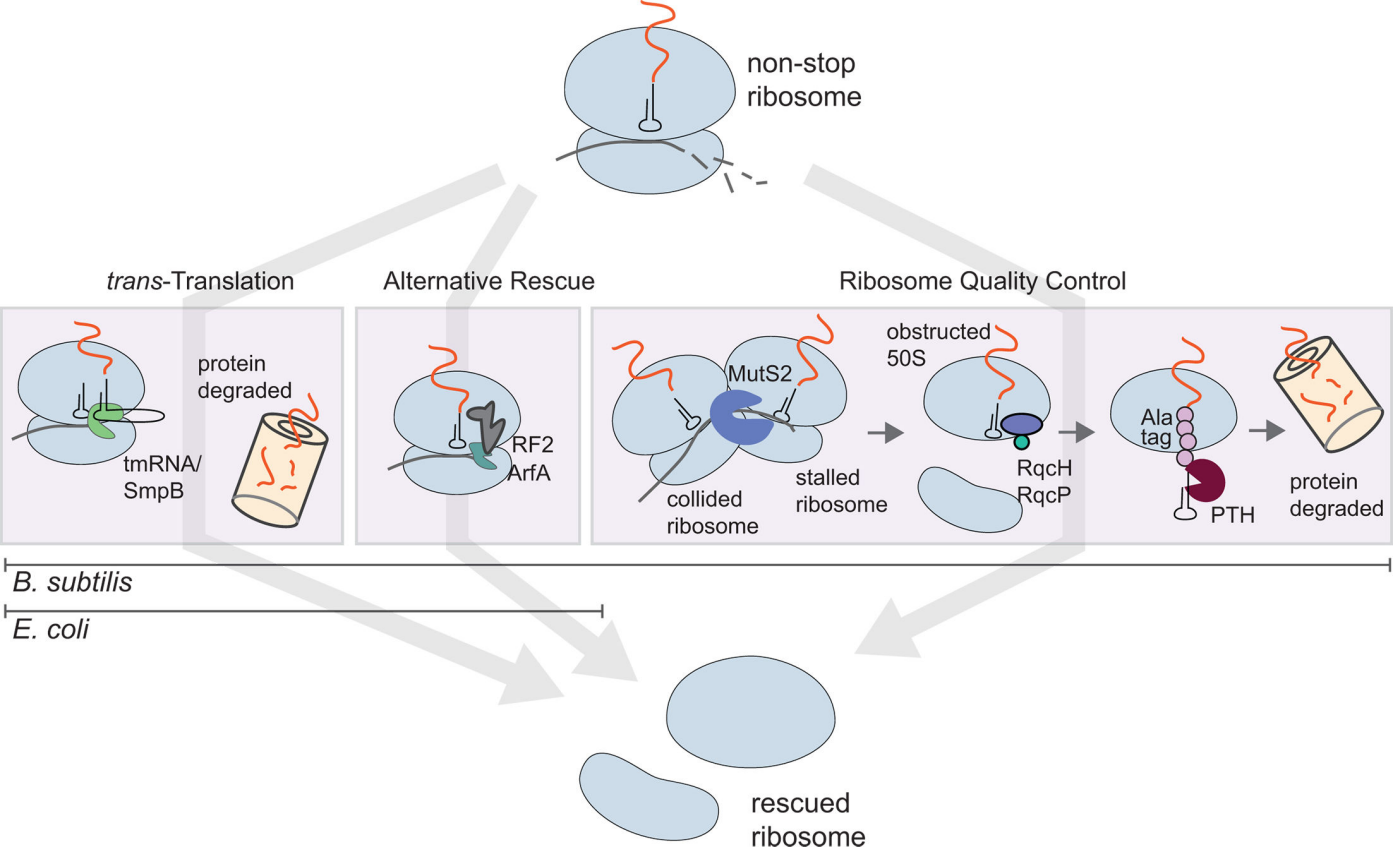
Summary of ribosome rescue pathways that are essential for viability. *trans*-Translation and ArfA are present in *E. coli*. Although ArfB is present in *E. coli*, it is not sufficiently expressed from the native locus to rescue viability and is therefore not shown. *B. subtilis* additionally uses the RQC pathway. Stalled ribosomes are recognized by a ribosome splitting factor such as MutS2 when collision occurs with the trailing ribosome. After being split from the mRNA, the large subunit remains obstructed with peptidyl-tRNA. RqcH and RqcP add an alanine tag to the stalled peptide, exposing the aminoacyl bond to the cytoplasm where it is cleaved by PTH.

Previous work demonstrates that bacteria can survive without *trans*-translation only if they encode one or more alternative non-stop rescue factors, either ArfA or ArfB (13, 15, 19). However, these studies were restricted mainly to Proteobacteria that lack the recently discovered ribosome rescue factor RqcH and the RQC pathway. RqcH is highly conserved in bacterial phyla outside of Proteobacteria (Fig. 1). Therefore, we investigated the essentiality of the non-stop rescue factors in *B. subtilis*, a model Firmicute that encodes the RQC pathway. We found that *B. subtilis* lacking all the canonical non-stop rescue factors (*trans*-translation, ArfA, and ArfB) can survive because it encodes RQC (Fig. 4 and 6). Moreover, expression of *B. subtilis* RqcH and RqcP in *E. coli* rescued the well-documented synthetic lethality of ∆*ssrA*∆*arfA* in this species (Fig. 2). These results support a role for the RQC pathway in rescuing non-stop ribosomes in diverse bacteria. Altogether, our work offers insight into non-stop mRNA rescue mechanisms across the domain bacteria and highlights the interchangeability of diverse rescue systems. However, many important questions remain especially with regard to why various phyla evolved to favor particular rescue pathways and why *trans*-translation remains universally conserved despite other non-stop rescue mechanisms being sufficient to support viability.

*trans*-Translation was the first non-stop rescue pathway to be discovered (5). The genes encoding *trans*-translation are present in nearly all bacterial genomes (>97%) (Fig. 1, Table 1). The high degree of *trans*-translation conservation when other non-stop rescue pathways are sufficient to support viability suggests that *trans*-translation is the most efficient means of non-stop rescue. Additionally, these findings suggest that the selective advantage imparted by *trans*-translation may extend beyond ribosome rescue. For example, *trans*-translation affects many facets of cellular physiology, such as regulating the transition from G1 to S phase in *C. crescentus* (42) and the expression of specific transcription factors in *E. coli* (43), and controlling pro-phage induction (44). More work is needed to identify the specific targets of *trans*-translation in diverse organisms to increase our understanding of how *trans*-translation impacts cellular physiology beyond the rescue of non-stop ribosomes.

The RQC pathway has some advantages over *trans*-translation for ribosome rescue. First, the RQC pathway does not require the mRNA on the stalled ribosome to be truncated. RqcH is agnostic to the source of the obstructed ribosomes it rescues, and therefore RQC is a more versatile rescue system. Moreover, unlike *trans*-translation, RQC does not require translation to be active to mediate rescue, a feature that may be important under conditions such as exposure to ribosome-targeting antibiotics or during the stringent response when translation is inhibited. We therefore propose that RQC is not a true backup mechanism for *trans*-translation, but has evolved to deal with diverse substrates, including non-stop ribosomes.

We did not detect RqcH in the 6,080 Proteobacteria we surveyed (Table 1), although a recent survey that included 15,204 Proteobacterial genomes detected RqcH in 0.14% of these genomes (27). How has the absence of RQC from most Proteobacteria impacted the evolution of ribosome rescue in these organisms? Interestingly, Gammaproteobacteria encode SmrB, an SMR domain-containing protein which binds to collided ribosomes but does not split them (29). Instead, SmrB possesses endonuclease activity that cleaves mRNA between collided ribosomes. *trans*-Translation, ArfA, and ArfB all require truncated mRNA, and their rescue activity is undetectable on ribosomes that are stalled mid-message (38, 39, 45, 46). Therefore, in the absence of RqcH, cells are more reliant on *trans*-translation and ArfA or ArfB to rescue ribosomes, and it becomes more important for mRNA cleavage to occur. Thus, loss of RqcH could be the pressure that was selected for the nuclease activity of SmrB.

The RQC pathway is highly conserved in Firmicutes. RqcH is present in >90% of the 2,633 Firmicute genomes that we surveyed. While the mRNA cleavage activity of SmrB has likely selected for *trans*-translation to be the dominant ribosome rescue pathway in *E. coli*, *B. subtilis* has evolved to rely heavily on RqcH. It is notable that the fitness defects of the ∆*smpB*∆*rqcH* strain in *B. subtilis* are even more severe than in ∆*smpB*∆*brfA* in liquid culture (Fig. 3). This finding indicates that obstructed 50S ribosomal subunits are a major problem for this organism and that ribosomes may be more likely to stall mid-message rather than on truncated mRNA.

RqcH does not act directly on non-stop ribosomes but requires that the ribosomal subunits be separated from the mRNA. Recently, MutS2 was identified as a ribosome splitting factor that can remove stalled ribosomes from mRNAs (29). Since MutS2 generates substrates for RqcH, we determined how frequently MutS2 and RqcH co-occur in bacterial genomes. We found an extremely high co-occurrence of MutS2 and RqcH in genomes across the bacterial domain (Fig. 5). However, experimentally, we found that RqcH does not have a strict requirement for MutS2 (Fig. 5). SmpB depletion from ∆*brfA*∆*mutS2* cells exhibited decreased fitness, but not as severe as depletion from ∆*brfA*∆*rqcH* cells (Fig. 5). Moreover, RqcH/RqcP were sufficient to rescue viability of ∆*arfA*∆*ssrA* in *E. coli*, a species that does not encode MutS2 (Fig. 2). Indeed, during revision, HrpA was identified as a ribosome splitter that could take the place of MutS2 in *E. coli* (47).

Why encode multiple mechanisms to rescue non-stop ribosomes? It is clear from decades of work on ribosome rescue that cells need at least one rescue pathway and there are likely conditions under which one rescue pathway is preferred over another. For example, a protein-based rescue system rather than an RNA-based rescue system may be preferred if cells are targeted by a nuclease toxin capable of cleaving tmRNA. Similarly, conditions that inhibit translation such as antibiotic treatment, the stringent response, and cold shock would also necessitate the use of the alternative rescue or RQC pathways since these pathways do not require rounds of translation elongation for rescue, unlike *trans*-translation. Finally, as the ribosome is a major target of antimicrobials and secondary metabolites produced by competing organisms in nature, it is likely that these pathways can be inhibited or modulated by neighboring bacteria in the environment. Thus, it is important to maintain the essential ribosome rescue function of multiple pathways even though the precise substrates of each pathway may vary.

## MATERIALS AND METHODS

### Strains and media

Strains are listed in Table 2 and were derived from *Bacillus subtilis* 168 *trpC2* and grown in lysogeny broth (LB) (10 g tryptone, 5 g yeast extract, 5 g NaCl per liter) at 30°C or 37°C with aeration where indicated. Gene deletions were made by transforming genomic DNA from the BKK collection (36) into the lab’s naturally competent wild type. The pDR244 plasmid was used to create unmarked ∆*smpB* and ∆*brfA* strains. Final concentrations of antibiotics used for selection included 100 μg/mL ampicillin, 20 μg/mL kanamycin, 100 μg/mL spectinomycin, 5 μg/mL chloramphenicol, and 1× MLS (macrolides/lincosamides/streptogramines, 1 μg/mL erythromycin and 25 μg/mL lincomycin).

### Growth curves for *B. subtilis*

Cells were grown overnight in LB with aeration and back diluted to a starting OD600 of 0.05. The cells were grown for 24 h at 37°C, shaking at 2 mm amplitude using Thermo Scientific 96-well flat bottom plates (cat. no. 167008). OD600 values were obtained in 15 min intervals from the BioTek Synergy H1 microplate reader, Gen5 3.11. Growth rates were measured using non-linear regression of logistic growth, and statistical analysis was performed using an unpaired *t*-test followed by a Welch’s correction using GraphPad Prism v10.1.1.

### Plasmid construction

Primers are listed in Table 2. *rqcH* from *Bacillus subtilis* was amplified using primers KC53 and KC54 and gel extracted. pBAD322 digested with EcoRI/NcoI was used as the plasmid backbone for Gibson assembly (48) of the PCR product resulting in pKC353. An IDT gblock containing the *rqcP* sequence with homology to pKC353 was then Gibson assembled after pKC353 digestion with NcoI/XbaI to create pKC426 (Genbank ID PQ015303). Plasmids were transformed into DH5alpha cells and selected on ampicillin plates containing 1% arabinose. Whole plasmid sequencing was performed by Plasmidsaurus using Oxford Nanopore Technology.

### CRISPR interference

To create the sgRNA targeting *smpB*, we amplified pJMP2 (37) using primer sp60 (22) and reverse primer HRH175 (33). The Phusion PCR product was Dpn1 treated for 2.5 h at 37°C followed by polynucleotide kindase (PNK) treatment for 1 h. The resulting band was gel extracted and ligated overnight at room temperature using T4 DNA ligase (NEB). pKC349 was transformed into DH5alpha and selected on ampicillin, and sequencing was performed by Plasmidsaurus. Genomic DNA of *B. subtilis* harboring dCas9 under a xylose-inducible promoter at *lacA* (37) was transformed into ∆*rqcH::kan,* ∆*brfA,* and ∆*brfA*∆*rqcH::kan* cells. pKC349 was ScaI digested to linearize and transformed into the above backgrounds for integration into *amyE*. Strains were grown in LB for 4 h at 37°C and back diluted to a final OD600 of 0.05 in 1× Tbase + 1 mM MgSO_4_. The cultures were serially diluted, and 10 μL was spotted onto LB agar containing 1% xylose and incubated at 30°C or 37°C overnight.

### Transduction

Phage lysate was obtained from *E. coli* ∆*arfA::kan*. MG1655∆*ssrA::cat* was electroporated with pKC353 or pKC426 and grown on LB agar containing ampicillin. Recipient cells were transduced with phage lysate (49–54) and selected on media containing 20 µg/mL kanamycin, 100 µg/mL ampicillin, and 1% arabinose to induce expression of RqcH (pKC353) and RqcH with RqcP (pKC426).

### Growth curves for *E. coli*

Cells were grown overnight in LB, and strains harboring the p*rqcH-rqcP* plasmids were grown with 100 µg/mL ampicillin and 1% arabinose to maintain the plasmid. The overnight cultures were back diluted to a starting OD600 of 0.05. The cells were grown in the presence of 1% arabinose or 1% glucose where indicated for 24 h at 30°C or 37°C, shaking at 2 mm amplitude using Thermo Scientific 96-well flat bottom plates (cat. no. 167008). OD600 values were obtained in 15 min intervals from the BioTek Synergy H1 microplate reader, Gen5 3.11.

### Gene detection

Genomes were obtained from the NCBI Reference Prokaryotic Representative Genomes database which contains >18,000 representative genomes, a subset of RefSeq that includes approximately one representative genome per species (55). Genomes were filtered for CheckM scores of >80% completeness and <10% contamination (56). Genes were detected using HMMER v3.3 (nhmmer) (hmmer.org) with an E-value cutoff of 0.05. HMMER profiles were built using ≥5 genes as annotated by the NCBI Prokaryotic Genome Annotation Pipeline (55). At least one gene sequence from every taxon expected to have the gene based on a preliminary search was included. Query sequences for each gene are given in Table S2. In Alphaproteobacteria like *Caulobacter crescentus*, the *ssrA* gene is interrupted by an internal loop that is excised from the final RNA product (31). Thus, two separate HMMER profiles were built and searches were performed for *ssrA* to separately probe Alphaproteobacteria and other phyla. Proteins closely resembling *B. subtilis* RqcH are described in NCBI as “NFACT RNA binding domain-containing proteins.” Only genes for proteins with this description were used when building the *rqcH* HMMER profile. The domain architecture of MutS2 is similar to that of MutS1, but the homology is only shared over approximately 1,000 bp. Therefore, hits were filtered for a coverage of >1,250 bp to remove erroneous *mutS1* hits. Similarly, *arfB* shares approximately 100 bp of homology to *prfB*, so hits were filtered for a coverage of >150 bp. SMR domains in other proteins resemble the C-terminus of *smrB*, so hits were filtered for a coverage of >400 bp.

### Phylogenetics

16S rRNA sequences of all genomes were identified and acquired using BLAST v2.13.0 (57), aligned using MAFFT v7.453 (58), and applied to FastTree v2.1.11 (59) to infer a maximum likelihood tree. FastTree produces unrooted phylogenies, so trees were midpoint rooted using the phangorn v2.11.1 package (60). Representatives from each phylum were randomly selected and used to subset the built 16S tree. The identities of the randomly selected genomes can be found in Table 2. Taxonomic classification was assigned to genomes using the NCBI Taxonomy database (61) and taxonkit v0.17.0 (62). Phyla were named using the conventions in Coleman et al. (63). The tree was visualized using ggtree v3.6.2 (64).

## Data Availability

Genome accession numbers and presence or absence of each rescue factor can be found in Table S1. Gene sequences used to build HMMER profiles are found in Table S2. The sequence for pKC426 is available in Genbank (ID PQ015303). Scripts for data acquisition and analysis can be found at https://github.com/cassprince/Ribosome_quality_control_conservation.

## SUPPLEMENTAL MATERIAL

Captions mbio.02322-24-s0001.docx Captions for Tables S1 and S2.

Table S1 mbio.02322-24-s0002.csv Genome assemblies and presence of rescue factors.

Table S2 mbio.02322-24-s0003.csv Gene query sequences.

ASM does not own the copyrights to Supplemental Material that may be linked to, or accessed through, an article. The authors have granted ASM a non-exclusive, world-wide license to publish the Supplemental Material files. Please contact the corresponding author directly for reuse.
