# Supplementary material for: The ribosome-associated quality control pathway supports survival in the absence of non-stop ribosome rescue factors
Source: mBio. 2024 Nov 13;15(12):e02322-24. doi: 10.1128/mbio.02322-24 (PMC11633108; doi:10.1128/mbio.02322-24)
Supplement: Captions — for Tables S1 and S2. [file mbio.02322-24-s0001.docx]

**Supplemental table legends**

**Table S1.** Accession numbers of genomes that were searched for the presence or absence of *ssrA*, *smpB*, *arfA*, *arfB*, *smrB*, *rqcH*, and *mutS2*. Gene presence is indicated as TRUE.

**Table S2.** Gene sequences of *ssrA*, *smpB*, *arfA*, *arfB*, *smrB*, *rqcH*, and *mutS2* that were used to build HMMER profiles.
